# Curcumin-Loaded Liposome Preparation in Ultrasound Environment under Pressurized Carbon Dioxide

**DOI:** 10.3390/foods11101469

**Published:** 2022-05-18

**Authors:** Wahyudiono, Jiayang He, Xin Hu, Siti Machmudah, Keiji Yasuda, Seiichi Takami, Hideki Kanda, Motonobu Goto

**Affiliations:** 1 Department of Materials Process Engineering, Nagoya University, Nagoya 464-8603, Japan; wahyudiono.c8@mso.tohoku.ac.jp (W.); he.jiayang@h.mbox.nagoya-u.ac.jp (J.H.); hu.xin@i.mbox.nagoya-u.ac.jp (X.H.); takami.seiichi@material.nagoya-u.ac.jp (S.T.); goto.motonobu@material.nagoya-u.ac.jp (M.G.); 2 New Industry Creation Hatchery Center, Tohoku University, 6-6-10 Aoba, Aramaki, Aoba-ku, Sendai 980-8579, Japan; 3 Department of Chemical Engineering, Institut Teknologi Sepuluh Nopember, Surabaya 60111, Indonesia; machmudah@chem-eng.its.ac.id; 4 Department of Chemical Systems Engineering, Nagoya University, Nagoya 464-8603, Japan; yasuda.keiji@material.nagoya-u.ac.jp; 5 Super Critical Technology Centre Co., Ltd., Kuwana 511-0838, Japan

**Keywords:** supercritical, curcumin, encapsulation, liposome, phospholipid

## Abstract

Curcumin-loaded liposomes were prepared using a supercritical carbon dioxide (SCCO_2_)–ultrasound environment system. The experiments were performed at temperatures of 40–70 °C and pressures of 10–25 MPa in a batch system with ultrasonication for 60 min. Transmission electron microscopy (TEM) images revealed liposome products with spherical morphologies and diameters of <100 nm. Dynamic light scattering (DLS) analysis indicated that the curcumin-loaded liposome nanosuspension exhibited good stability. Changing the operating conditions influenced the amount of liposome-encapsulated curcumin; as the operating temperature or pressure increased, the diameter of the liposome products and the amount of liposome-encapsulated curcumin increased and decreased, respectively. Herein, we described an innovative and practical organic-solvent-free method for generating liposomes from phospholipids.

## 1. Introduction

*Curcuma longa* L. (turmeric) is a plant that is native to tropical South Asia; it is widely cultivated in subtropical and tropical zones. This rhizomatous herbaceous perennial plant is generally used in production of a natural colorant, namely curcumin. This chemical compound, as the main active substance of turmeric, has a low-molecular-weight polyphenol that is responsible for the color of turmeric (bright yellow). Curcumin is utilized in the pharmaceutical field owing to its anti-inflammatory, antimicrobial, antibacterial, antifungal, and anticarcinogenic properties [1]. Several studies have reported that this compound can be used to treat various diseases, such as psoriasis, human immunodeficiency virus (HIV), multiple myeloma, myelodysplastic syndrome, and Alzheimer’s disease [2,3,4]. However, curcumin exhibits high hydrophobicity and poor solubility in water, and has a complex structure. In addition, it possesses poor absorption, rapid metabolism, and rapid systemic elimination, which may limit its use in functional food development and pharmaceutical applications [5,6,7]. Therefore, curcumin must be modified to prevent degradation and improve aqueous dispersion, resulting in increased utilization in food and pharmaceutical applications [5,8,9].

Liposomes are spherical vesicles comprising one or more phospholipid bilayers that are surrounded by an aqueous compartment. Liposomes are involved in various therapeutic (hydrophobic and hydrophilic) diagnostic agents that can protect the encapsulated agent from metabolic processes and provide a higher drug payload per particle. Owing to their unique properties, liposomes are employed in drug delivery systems. Typically, sphere-shaped vesicles have sizes ranging from 25 nm to several micrometers. Based on the lamellae number and size, liposomes are grouped as follows: multivesicular vesicles (MVVs), large multilamellar vesicles (LMVs), oligolamellar vesicles (OLVs), giant unilamellar vesicles (GUVs), large unilamellar vesicles (LUVs), medium-sized unilamellar vesicles (MUVs), and small unilamellar vesicles (SUVs). The sizes for MVVs, LMVs, OLVs, GUVs, and LUVs range from a few hundred nanometers to several micrometers, whereas the sizes for MUVs and SUVs range from 100 to 500 nm and 20 to 100 nm, respectively [10]. Of these, MUVs and SUVs are the most thermochemically sensitive. They are often employed as pharmaceutical drug carriers [11].

The conventional method of liposome production has tremendous limitations; it requires an organic solvent, the process is a complex, multistage process, the process is costly due to the high energy consumption, and the formed liposomes mostly exhibit poor stability [12,13]. In this study, a supercritical carbon dioxide (SCCO_2_)–water mixture, operated in an ultrasound environment, was employed as a medium to generate organic-solvent-free liposomes. Curcumin was simultaneously encapsulated with the formed liposomes. Carbon dioxide is generally recognized as a safe (GRAS) solvent, and most solvents are employed under supercritical conditions. The critical point of CO_2_ is relatively low (critical temperature, *T*_c_ = 31.06 °C; critical pressure, *P*_c_ = 7.38 MPa), which may prevent thermal deterioration during the process. When SCCO_2_ is employed as a medium for liposome generation, it may favor amphiphilic aggregation into amphiphilic nanoforms owing to its unique properties [14]. SCCO_2_ possesses low interfacial tension, high diffusivity, low viscosity, and tunable density, which cannot be easily achieved by other solvents (organic solvents). CO_2_ is not only readily available, inexpensive, nonflammable, tasteless, odorless, inert, and environmentally friendly, but can also be easily separated from the product under ordinary conditions; hence, it does not result in product contamination.

There are several methods to decrease liposome size, including homogenization, extrusion, and sonication. Batch and probe-type sonication are easy and common methods to decrease liposome size [12]. In the sonication technique, high energy is applied to the medium, where lipid vesicles disperse to shift the larger liposomes (LMVs) into smaller liposomes (SUVs). In probe-type sonication, the sonicator tip directly contacts the medium containing the lipid vesicle; therefore, there is potential contamination of the metal element from the probe device because the metal element of the probe may slough off and release into the medium during sonication [15]. To prevent contamination by the metal element probe, here, batch-type sonication was applied to the SCCO_2_–water mixture as a medium for liposome generation to decrease the liposome product size. Furthermore, this study aimed to utilize the SCCO_2_–water mixture system in an ultrasound environment to generate smaller liposomes and simultaneously encapsulate curcumin with the formed liposomes.

## 2. Results and Discussion

**Figure 1:**
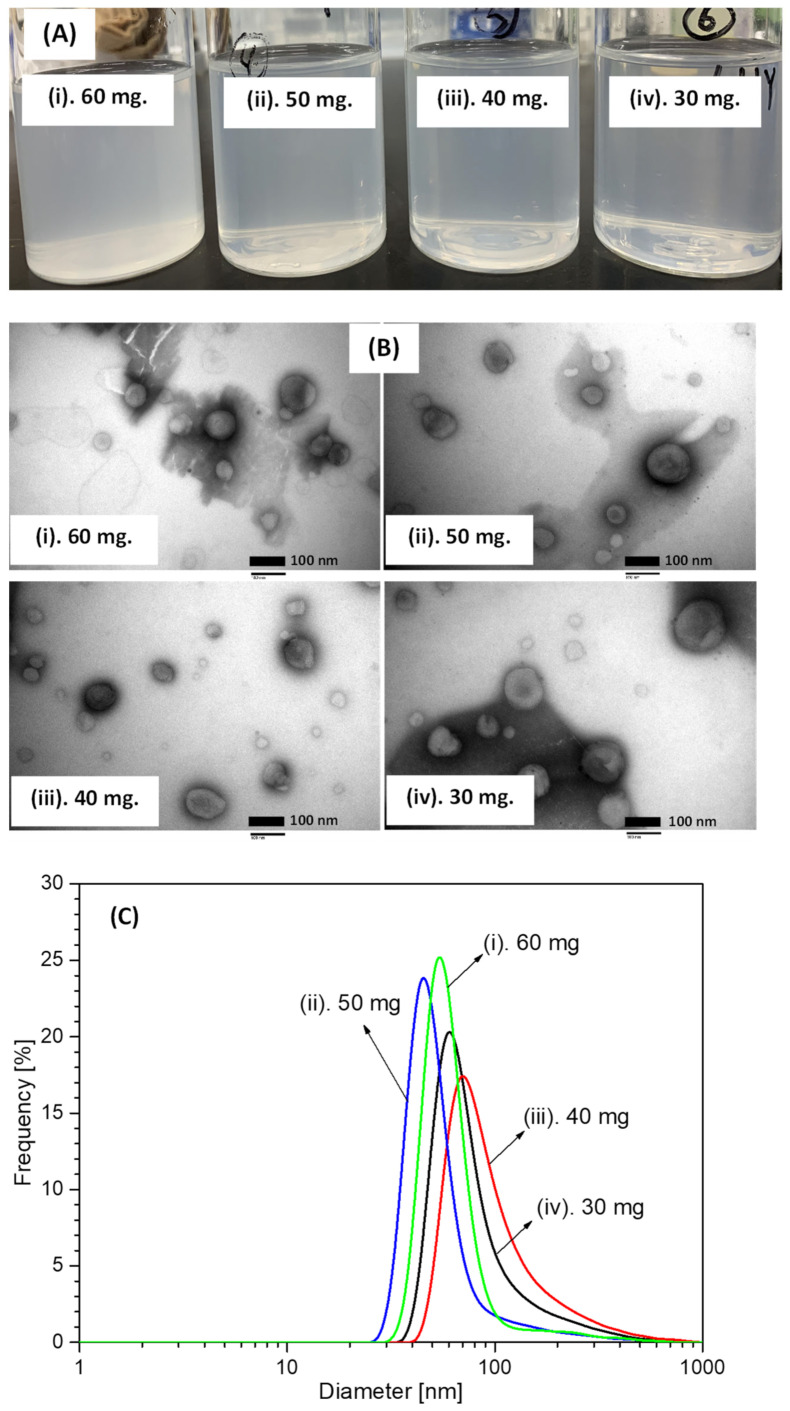
Photographs of liposome dispersions prepared at a temperature of 50 °C and pressure of 10 MPa (**A**), with their TEM images (**B**) and size distributions (**C**).

Figure 1A shows a photograph of the collected aqueous solution products obtained after hydrogenated soy phosphatidylcholine at various concentrations was loaded in a stainless steel (SUS) vessel and dispersed in a liquid water medium under SCCO_2_ conditions at 50 °C and 10 MPa with 45 kHz ultrasound irradiation. At each amount of loaded hydrogenated soy phosphatidylcholine, the collected aqueous solution products exhibited similar colors (opaque–transparent). In principle, liposomes can be formed as spherical phospholipid liquid crystalline phases via the dispersion of phospholipids in a liquid water medium by shaking while supplying sufficient energy. Hence, liposomes were generated when hydrogenated soy phosphatidylcholine and distilled water were loaded into the SUS vessel under pressurized CO_2_, and then ultrasound irradiation was applied to provide sufficient energy input. This phospholipid arranged itself into bilayer vesicles and multilayer structures comprising lipid bilayers. The thickness and length of bilayer vesicles may increase owing to an ultrasound irradiation process [16,17,18]. CO_2_ may simultaneously infiltrate and dissolve in a liquid water medium, resulting in a dissociation equilibrium forming carbonic acid, and the dehydration reaction might occur on the remaining CO_2_ molecule [19,20]. This dissociation equilibrium may enhance the interaction between hydrogenated soy phosphatidylcholine, mainly in the fatty acyl chain, and CO_2_-derived molecules. When CO_2_ depressurization was performed to release pressure from 10 MPa to ambient pressure, CO_2_ molecules quickly flowed out of the phosphatidylcholine, yielding highly dispersed liposomes. As a result, as shown in Figure 1A, the color of the solution changed from transparent to opaque.

In addition to generation of small and large unilamellar liposome products, in this study, liposomes with multivesicular and multilamellar structures were also generated. Transmission electron microscopy (TEM) was used to characterize the morphology and lamellarity of the liposome products. The TEM technique is widely utilized for the characterization of nanodelivery systems. Figure 1B depicts TEM images of the liposome products generated from hydrogenated soy phosphatidylcholine under SCCO_2_ conditions at 50 °C and 10 MPa with 45 kHz ultrasound irradiation. As depicted in this figure, the morphology of the liposome generation products was spherical or sphere-like at each concentration of loaded hydrogenated soy phosphatidylcholine. This morphology was due to liposomes comprising spherical or circular bundle structures as the main structures; however, they can also comprise concave or oblate circular bundle structures [21,22]. Frederik and Hubert reported that liposomes generally appear as unique ring-shaped structures in TEM micrographs because of the relatively high contrast of hydrogenated soy phosphatidylcholine structures [21]. They also reported that solute concentration did not influence liposome morphology, but implied increasing liposome numbers in the aqueous solution medium. Consequently, the liposome generation products possessed a spherical morphology, although different amounts of hydrogenated soy phosphatidylcholine were loaded into the SUS vessel as a starting material (see Figure 1B). 

Photographs of the collected aqueous solution products and their TEM images revealed that the various amounts of loaded hydrogenated soy phosphatidylcholine did not shift the physical properties of the liposome generation products, i.e., shape and size. The size of the liposome generation product was determined by measuring the solution products using a wet laser diffraction sizing (DLS) device, which is the most commonly used tool to measure emulsion system particles between 3 nm and 3 µm [23]. Notably, the liposome size is a critical parameter for nanosuspension characterization because it significantly impacts its biodistribution and activity. Hence, it was necessary to carefully measure the size of the generated liposome products. Figure 1C illustrates the size distribution of the liposome products obtained from hydrogenated soy phosphatidylcholine at various concentrations. The generated liposome product possessed a single peak at each concentration with a size range of 30–400 nm, predominantly at around 40–100 nm. Liposome size and dispersity are generally affected by the concentration of phospholipid as a starting material; the liposome size increases as the amount of loaded phospholipid increases [13,24]. Wright and Burgess reported that liposome size and dispersity, when generated by the injection technique, depended on the concentration of phospholipid in the medium. They reported that a larger amount of phospholipid injection in the medium resulted in a larger dispersity due to the different sizes of phospholipid droplets were formed [13]. The same tendency was also found when Sakdiset et al. conducted experiments on phospholipid selection to design a liposome preparation with high skin-penetration-enhancing effects [24]. However, as shown in Figure 1C, increasing the amount of hydrogenated soy phosphatidylcholine from 30 to 60 mg did not seem to have a significant impact on the size of the liposome products. Hydrogenated soy phosphatidylcholine loaded at 50 mg yielded the smallest size. At this load, the bilayer structure of the liposomes probably underwent rearrangement, resulting in smaller liposome sizes in higher quantities. This result also indicated that better size distribution occurred under these conditions [25]. Thus, 50 mg of loaded hydrogenated soy phosphatidylcholine was used as the starting material for liposome formation experiments with curcumin addition in an ultrasound environment under pressurized CO_2_.

**Figure 2:**
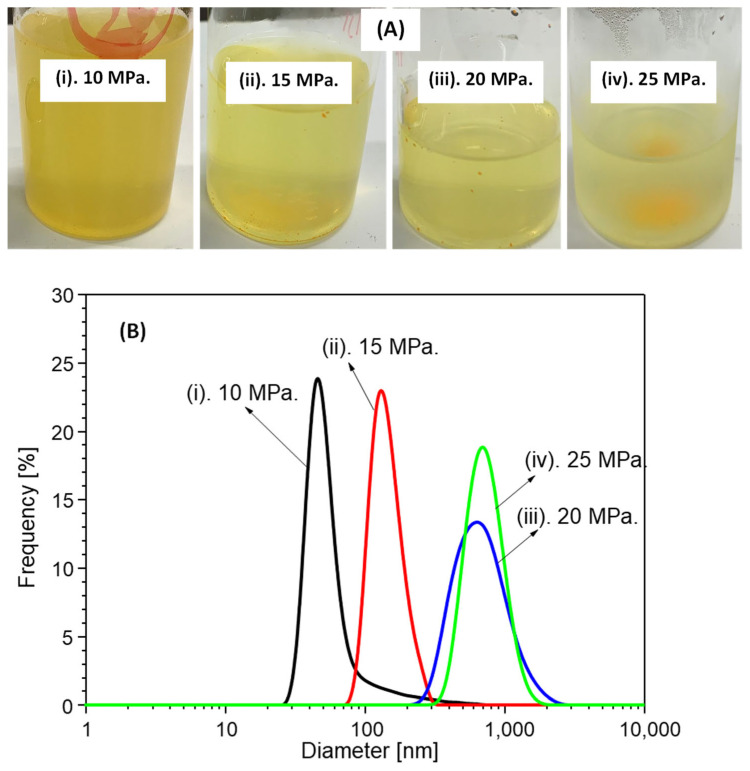
Aqueous suspensions (**A**) and their size distributions (**B**). Suspensions were prepared at 50 °C and various operating pressures.

Figure 2 shows a photograph of the aqueous suspensions (a) and their size distributions (b) obtained after 50 mg of hydrogenated soy phosphatidylcholine and 20 mg of curcumin were loaded into the SUS vessel and dispersed in a liquid water medium under SCCO_2_ conditions at 50 °C and various operating pressures with 45 kHz ultrasound irradiation. The aqueous suspension product had a light transparent yellow color at each operating pressure. As mentioned above, liposomes are spherical vesicles that can be generated by the interactions of phospholipids and liquid water because phospholipid molecules possess naturally amphiphilic features, where the phospholipid lipophilic side generally faces internally and the hydrophilic side faces externally to the liquid water medium. Therefore, liposomes may integrate both hydrophilic and hydrophobic bioactive compounds, including curcumin. Therefore, liposomes were spontaneously generated from hydrogenated soy phosphatidylcholine when loaded and dispersed in the liquid water medium. Simultaneously, the generated liposomes may interact and encapsulate curcumin when hydrogenated soy phosphatidylcholine and curcumin are loaded and dispersed in the same liquid water medium. This incorporation may occur on the polar head group of a lipid bilayer through hydrogen bonding [26,27,28]. As a result, as shown in Figure 2A, curcumin powder was easily dispersed into the liquid water medium, resulting in a light transparent yellow color. 

These aqueous suspensions were investigated by DLS to measure the sizes of the liposomes containing curcumin; based on these findings, the particle size distribution of in the aqueous suspension products seemed to possess a single peak diameter under each experimental condition. This shows that the nanosuspension particle size was in the wide range of 40–800 nm; the smallest particle size (around 40 nm) was observed when the experiment was performed at 10 MPa. It is well known that the dissolution of CO_2_ molecules in a liquid water medium may promote phospholipid dispersion to generate a nanosuspension, where, at the same operating temperature, increasing operating pressures enhance the dissolution of CO_2_ molecules in the liquid water medium. CO_2_ molecules interfered with phospholipid molecules, resulting in enhanced dispersion, leading to the generation of nanosuspensions with smaller particle sizes (<100 nm). Hence, the dissolution of CO_2_ molecules in the liquid water medium was expected to enhance the dispersion of hydrogenated soy phosphatidylcholine, resulting in the generation of nanosuspension particles with diameters smaller than 100 nm. At the same time, the application of ultrasound irradiation in the SCCO_2_–liquid water system may also have enhanced CO_2_ molecule dissolution because the acoustic vibration of ultrasound irradiation can impose CO_2_ molecule mass transfer into liquid water [29,30,31]. As a result, an intense dispersion of hydrogenated soy phosphatidylcholine occurred, which improved the production of nanosuspensions with smaller particle sizes.

However, as shown in Figure 2B, although the operating temperature of 50 °C favored the flexibility and mobility of hydrogenated soy phosphatidylcholine in the liquid water medium to generate smaller liposomes with better uniformity [31], increasing the operating pressure from 10 MPa to 25 MPa under the same operating conditions (50 °C with 45 kHz ultrasound irradiation) did not decrease the particle sizes in the nanosuspension. Conversely, increasing the operating pressure from 10 to 25 MPa seemingly promoted an increase in the particle size of the nanosuspension. Although the increasing operating pressure might have facilitated and enhanced the quantity of CO_2_ molecules in the liquid water medium, resulting in the uniform dispersion of hydrogenated soy phosphatidylcholine with smaller diameters, the aggregation of generated liposomes might also have occurred during CO_2_ depressurization at the end of each experiment [32]. Shashidhar and Manohar conducted experiments on liposome nanocharacterization to encapsulate water-soluble compounds under SCCO_2_, and reported that CO_2_ depressurization at a slow rate (around 25 bar/min) may better control the size and size distribution of liposomes [32]. However, in this study, the CO_2_ depressurization rate was not observed in each experiment. In addition to the CO_2_ depressurization rate, the size of the particle products might also have been influenced by the presence of curcumin in the liquid water medium. Under CO_2_ conditions, Zhao et al. performed experiments on carotenoid encapsulation in liposomes using lutein as a carotenoid model compound [31]. They reported that increasing the operating pressure enhanced the quantity of lutein incorporated into the liposomes, resulting in a larger vesicle size. At higher operating pressures, the lutein monomer could fill the space and break the imperfect pack of bilayer structures generated by the phospholipid as a starting material. Next, the lutein hydrophobic side could interact with the soy lecithin molecules to confirm the ordering of phospholipid alkyl bonds and decrease the mobility and fluidity of the soy lecithin molecules. This might also have resulted in a larger vesicle size. The same phenomenon may have been observed when curcumin powder was loaded into the SUS vessel together with hydrogenated soy phosphatidylcholine and distilled water as a medium under pressurized CO_2_ and ultrasound irradiation conditions. Hence, as exhibited in Figure 2B, the size of the generated liposomes containing curcumin powder increased with increasing operating pressures.

**Figure 3:**
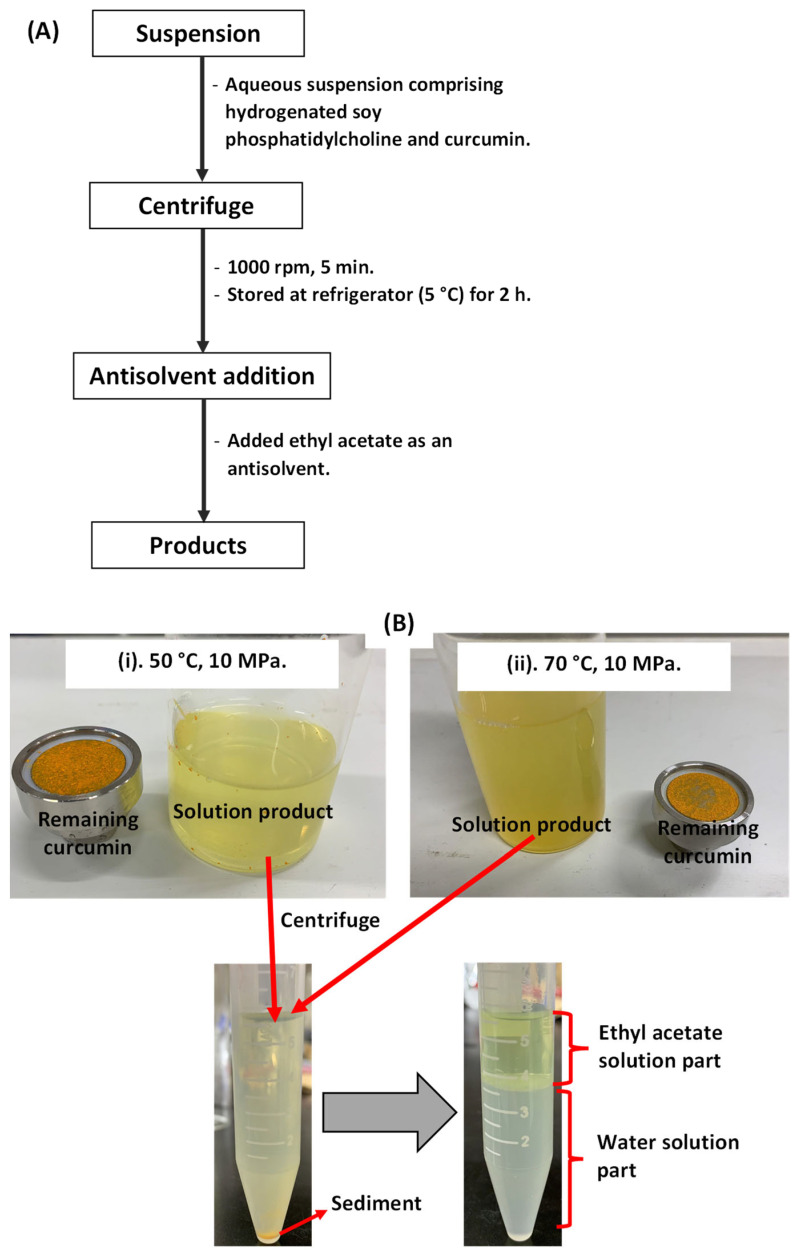
Curcumin separation process: (**A**) separation procedure and (**B**) photographs.

To determine the total amount of liposome-encapsulated curcumin, the collected aqueous solution products were injected into and analyzed using a high-performance liquid chromatography (HPLC) system. Prior to HPLC analysis, liposome-encapsulated curcumin was separated from the collected aqueous products. Figure 3A,B show the separation procedure for liposome-encapsulated curcumin from the collected aqueous products. First, the aqueous solution (10 mL) was loaded into a centrifuge bottle and sealed tightly. It was then centrifuged (CN-1040, Hsiangtai, New Taipei City, Taiwan) at 1000 rpm. After centrifugation (5 min), the supernatant was removed and stored in a refrigerator for 2 h. Next, the upper part of the supernatant (around 3 mL) was removed from the centrifuge bottle using a micropipette (Nichiryo, Nichipet EX Plus II, Tokyo, Japan) and loaded onto others. The same method was used for sample preparation of DLS and TEM characterizations. Here, a pure ethyl acetate solvent (around 2 mL) was employed as an antisolvent to extract liposome-encapsulated curcumin from the supernatant. After the mixture of the supernatant and ethyl acetate solvent was shaken well, it was left for 30 min. The ethyl acetate layer was then separated and transferred to a sample vial. The ethyl acetate solution containing curcumin was then injected into the HPLC device to determine the amount of liposome-encapsulated curcumin.

**Figure 4:**
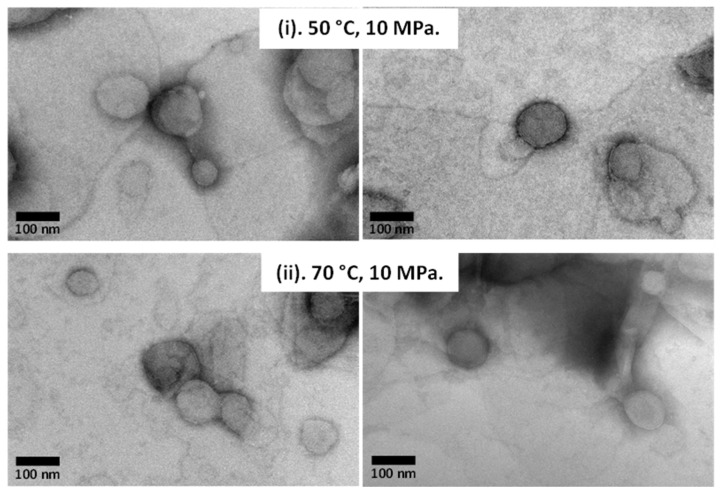
TEM images of liposomes containing curcumin.

In addition to the upper part of the supernatant being analyzed using HPLC, this part was also characterized by using a TEM device to observe the morphology and lamellarity of the generated liposome products containing curcumin. Figure 4 shows TEM images of the generated liposome products containing curcumin when the experiments were conducted at a constant operating pressure (10 MPa) and different operating temperatures (50 and 70 °C). Similar to the results presented above (Figure 1), the liposome products containing curcumin also possessed spherical or sphere-like properties at each operating temperature. This was a good result in terms of liposome morphology, where liposomes with spherical structures typically possess high thermodynamic stability [18,33,34]. Yu et al. investigated several post-processing methods to improve the stability of liposomes and reported that spherical morphology is an important property for final liposome products [34]. Zhao et al. also reported that liposome morphology is an important factor in manipulating the properties of liposomes for applications in nutraceutical and drug delivery systems [18]. They explained that an ideal liposome is one with a uniform size distribution and a spherical structure without any surface rupture or roughness. In addition to spherical structure being a morphological feature of liposomes, the spherical shape of liposomes with a high curvature also made it easier to achieve close contact between lipid bilayers [33,35]. Based on these results, the addition of curcumin did not have a significant effect on the original morphology of the liposomes generated from hydrogenated soy phosphatidylcholine.

**Figure 5:**
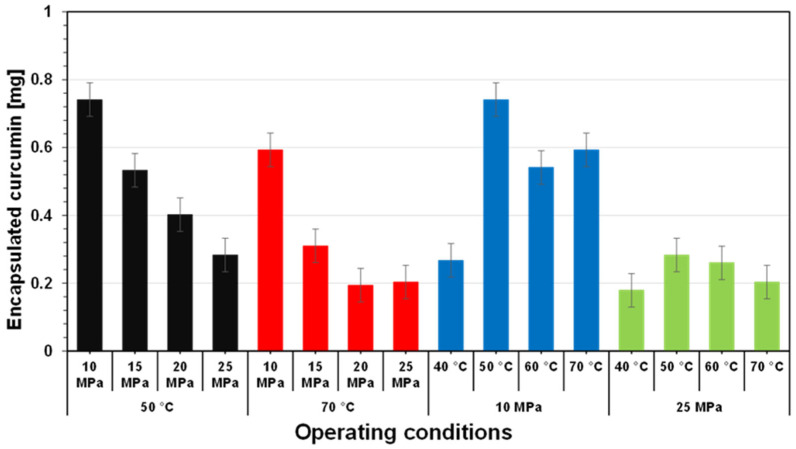
Amount of curcumin encapsulated under various operating conditions.

Figure 5 shows the amount of liposome-encapsulated curcumin in the collected aqueous products when the experiments were performed at various operating pressures at operating temperatures of 50 °C (black) and (B) 70 °C (red). At 50 °C, the amount of liposome-encapsulated curcumin decreased as the operating pressure increased from 10 MPa to 25 MPa. When the encapsulation experiment was conducted at an operating pressure of 10 MPa, the amount of liposome-encapsulated curcumin was approximately 0.74 mg. The amount of curcumin decreased significantly to 0.28 mg when the operating pressure of the encapsulation experiment increased to 25 MPa. The same phenomenon was observed when the encapsulation experiment was conducted at an operating temperature of 70 °C, with operating pressures ranging from 10 to 25 MPa. At an operating pressure of 10 MPa, there was 0.59 mg of liposome-encapsulated curcumin. This decreased significantly to 0.20 mg when the operating pressure increased to 25 MPa. As mentioned above, increasing the operating pressure at a constant operating temperature may improve the dissolution of CO_2_ molecules in the liquid water medium. This can interfere with phospholipid molecules more intensely, resulting in enhanced dispersion and the generation of a nanosuspension with small particle sizes. However, as shown in Figure 5, increasing the operating pressure at a constant operating temperature negatively impacted the amount of liposome-encapsulated curcumin; the amount of liposome-encapsulated curcumin decreased as the operating pressure increased. Perhaps, at excessive operating pressure, weakened interactions between the fatty acyl chains occurred during liposome preparation, thus disintegrating the liposome products and decreasing the amount of liposome-encapsulated curcumin [18,36]. Zhao et al. conducted experiments on the preparation of anthocyanin-loaded liposome in liquid water under pressurized carbon dioxide [18]. They reported that although the increase in operating pressure from 60 to 300 bar at a constant operating temperature significantly decreased the mean diameter of the liposome products, increasing operating pressure also significantly reduced the efficiency of encapsulation and the anthocyanin loading capacity. This is because increasing the operating pressure increased the accumulation of carbon dioxide molecules in the liquid water medium, leading to a weakened interaction between the fatty acyl chains. Dong et al. also reported that increasing the operating pressure at a constant operating temperature could disintegrate liposome products, leading to reduced encapsulation efficiency when experiments were conducted to prepare and characterize egg yolk immunoglobulin-loaded chitosan liposomes in a liquid water medium under pressurized carbon dioxide [36].

**Figure 6:**
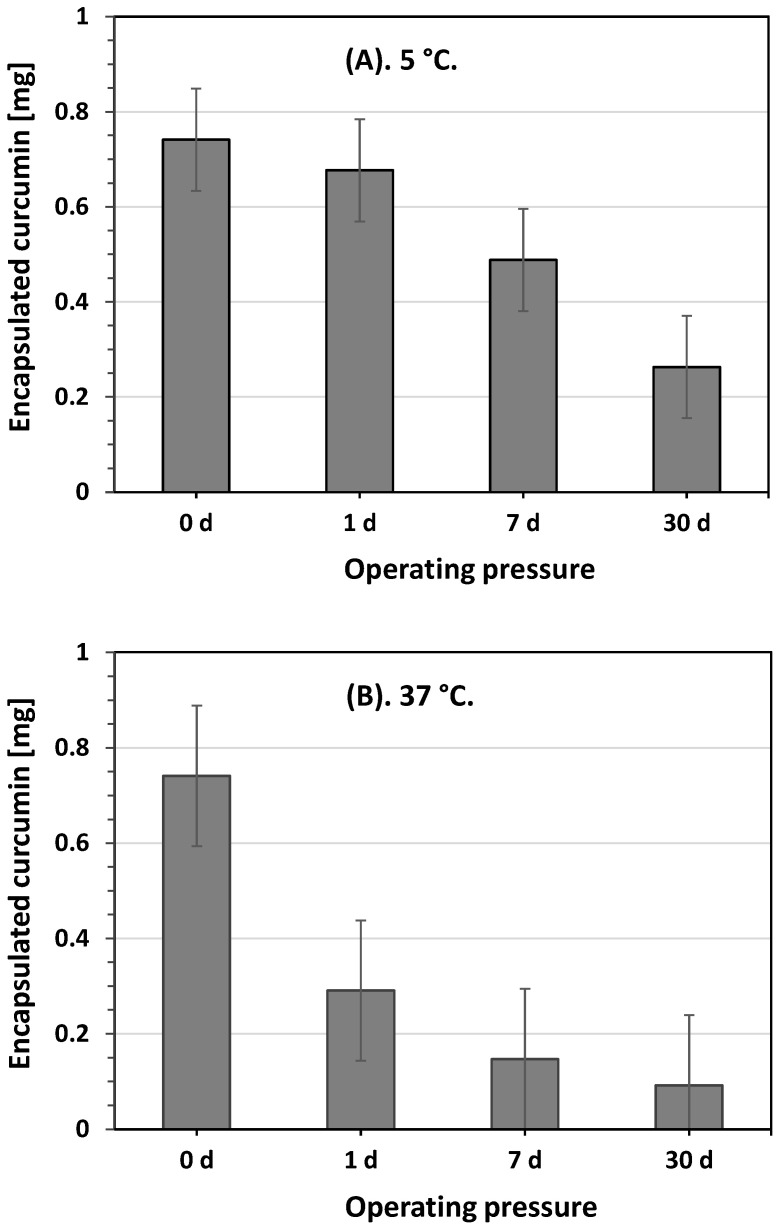
Amount of encapsulated curcumin after storage at temperatures of (**A**) 5 °C and (**B**) 37 °C.

In addition to the operating pressure, changing the operating temperature also impacted the amount of liposome-encapsulated curcumin following preparation by the SCCO_2_ technique. Figure 5 also shows the amount of liposome-encapsulated curcumin in the aqueous products when encapsulation was performed at various operating temperatures with operating pressures of 10 MPa (blue) and 25 MPa (green). As depicted in Figure 5 (blue), the amount of liposome-encapsulated curcumin was initially 0.27 mg when the experiment was performed at an operating temperature of 40 °C and an operating pressure of 10 MPa. At the same operating pressure, the curcumin amount increased to 0.74 mg at an operating temperature of 50 °C, and then decreased to 0.59 mg at 70 °C. The same phenomenon was found when curcumin encapsulation using liposomes was conducted at a constant operating pressure with various operating temperatures. At 40 °C, the amount of liposome-encapsulated curcumin was 0.18 mg. When the operating temperature was increased to 50 °C, the amount of liposome-encapsulated curcumin reached 0.28 mg. It then decreased to 0.20 mg at an operating temperature of 70 °C with the same operating pressure. Zhao et al. performed lutein encapsulation in liposomes under pressurized carbon dioxide and observed that the operating temperature had a significant effect on the physical features of aqueous suspensions [31]. Operating temperature affected bilayer membrane features such as permeability, fluidity, and degree of order. They explained that high operating temperatures (>50 °C) may help to break the interactions between the phospholipid alkyl chains, i.e., mainly van der Waals forces and hydrophobic interactions. Hence, increasing the operating temperature may favor the encapsulation of lutein because it may enhance the permeability, fluidity, and disorder of the bilayers. In contrast, in addition to the high operating temperature in the pressurized carbon dioxide system, which weakened the dispersion of phospholipid bilayers and encapsulated compounds, Shishir et al. reported that high environmental temperatures in an aqueous medium may cause a high energy input that can interfere with the partial interactions between phospholipids and encapsulated compounds, including lutein compounds [37]. Consequently, as shown in Figure 6 (blue and green), the amount of liposome-encapsulated curcumin decreased as the operating temperature increased at a given operating pressure.

One of the important steps in liposome generation processes involving the encapsulation of compounds with liposomes is to observe their characteristic features. This was used to confirm the desired liposomal products. The physical parameters of the liposome products (particle size and zeta potential) are summarized in Table 1. As a critical liposome parameter, liposome size may affect biodistribution and activity [33,35]. It can also affect drug release as well as transdermal and dermal drug delivery. The liposome size was analyzed by introducing the collected aqueous solution products into a DLS device, which is the most common device for analyzing nanomaterial size in suspensions, including liposomes generated from hydrogenated soy phosphatidylcholine. As shown in Table 1, the size of the liposome products increased with increasing operating pressure at each operating temperature. As previously mentioned, the increased operating pressure resulted in increased sizes of the liposome products, owing to coalescence or aggregation during depressurization. Although DLS is a rapid technique that can be used to measure particle sizes from 2 mm to <1 nm and requires a small amount of sample, this analysis technique has poor sensitivity to aggregation. The presence of aggregates in a sample solution, even in small amounts, may cause the particle diameter to increase. As a result, as listed in Table 1, liposome products with diameters larger than 500 nm were found under each operating condition. Conversely, liposome products with large diameters owing to liposome coalescence or aggregation were not observed in the TEM images (Figure 1B and Figure 4). The TEM images showed that the diameters of the liposome products were around 100 nm or below 100 nm. This distinction might be due to differences in the sample preparation and limitations of the nanoparticle sizing technique. The TEM device may act as a tool to capture the individual particle size, and can be employed to measure the particle size once the samples have been suspended and dried. Thus, larger sizes of the liposome products measured due to agglomeration can be minimized. Since DLS was conducted in solution form, the agglomeration and density of particles may have interfered with the reliability of the measurements during analysis. Therefore, the diameter of the liposome products determined by TEM was much smaller than that determined by DLS [38,39,40].

Similarly to the diameter size of liposomes, the zeta potential of liposomes is another important parameter that should be considered in liposome generation after the liposome preparation is complete. The zeta potential is the potential difference between the bulk dispersion medium in which the particles are dispersed and the slipping plane of the fluid attached to the dispersed particles. This parameter often reflects the liposome surface charge, which can be used to assist in the prediction of the liposome product’s stability. In other words, the zeta potential can be applied to represent the interaction between the biological environment and the stability of the colloidal dispersion. When the value of the zeta potential is low or near zero, there is no electrostatic repulsion between the liposome products. Accordingly, liposome product flocculation related to the instability of the suspension is generally due to the nature of the attractive force. Conversely, if the liposome suspension possesses a large positive or negative zeta potential, the aggregation of liposome products may not occur because they repel each other. A colloidal dispersion system is generally considered to be stable when its zeta potential value is in the range of 30 mV (either positive or negative) [41,42]. In this study, the suspension containing liposome products had a zeta potential value above 30 mV at each operating condition. This indicates that the suspension system comprised liposomes generated from hydrogenated soy phosphatidylcholine, and liposome-encapsulated curcumin was a stable colloidal dispersion system.

Similarly to other colloidal dispersion systems, liposomes may also undergo aggregation, phase changes, and fusion upon storage. Here, the liposome products containing the curcumin compound were stored in an incubator (37 °C) and a refrigerator (5 °C) for several days to monitor the liposome storage stability, because a stable formulation may physically maintain the quantity of encapsulated materials, including curcumin [32,43]. Next, as a function of the storage time, the amount of liposome-encapsulated curcumin was analyzed using HPLC. Figure 6 depicts the amount of liposome-encapsulated curcumin in the collected aqueous solution products when the encapsulation experiment was conducted at an operating temperature of 50 °C and pressure of 10 MPa. This showed that at each storage temperature, the amount of liposome-encapsulated curcumin decreased with prolonged storage time. However, the amount of liposome-encapsulated curcumin decreased faster at a storage temperature of 37 °C than that at a storage temperature of 5 °C over the same storage time. Initially, the amount of liposome-encapsulated curcumin was approximately 0.74 mg at each storage temperature for a 0-day storage period. After 1 d of storage, it decreased to 0.68 and 0.29 mg at storage temperatures of 5 °C and 37 °C, respectively. After 30 d of storage, this amount decreased significantly to 0.09 mg when the collected aqueous solution was stored at 37 °C. This indicates that, in addition to the increasing storage temperature, the interaction among phospholipid molecules was reduced, resulting in decreased liposomal system stability; increased storage temperature may also have improved phospholipid hydrolysis, causing liposome membrane defects and resulting in the release of encapsulated curcumin [44,45]. Jin et al. conducted an experiment on curcumin liposomes prepared using soybean lecithin and milk fat globule membrane phospholipids [44]. They reported that increasing the storage temperature improved liposome membrane permeability, resulting in leakage of encapsulated curcumin from the liposome interior. Conversely, the membrane of the liposomes was in a colloidal form at low storage temperatures, which prevented the high loss of curcumin from the interior of the liposomes. The same results were also reported by Pan et al., who observed the storage stability of astaxanthin-loaded liposomes [45]. They explained that the phospholipid hydrolysis rate increased with increasing storage temperature, resulting in an increased leakage of the encapsulated astaxanthin from the liposome products. Based on these results, a low environmental temperature (5 °C) is recommended for the storage of collected aqueous solution products in order to minimize curcumin leakage from liposome encapsulation.

## 3. Materials and Methods

### 3.1. Materials

Hydrogenated soy phosphatidylcholine (S–10 PLUS) phospholipid and curcumin (C_21_H_20_O_6_, 036-04922) were obtained from Nikko Chemicals Co., Ltd., Tokyo, Japan and Wako Pure Chemical Industries, Ltd., Osaka, Japan, respectively. Ethyl acetate (CH_3_COOC_2_H_5_, 99.0%) and acetonitrile (CH_3_CN, 99.5%) were purchased from Wako Pure Chemical Industries, Ltd., Osaka, Japan. Phosphotungstic acid (H_3_PW_12_O_40_, sc-215716) was purchased from Nacalai Tesque, Inc. (Kyoto, Japan). Carbon dioxide (CO_2_, 99%) was obtained from Tomoe Shokai Co., Ltd., Tokyo, Japan.

### 3.2. SCCO_2_–Ultrasound

**Figure 7:**
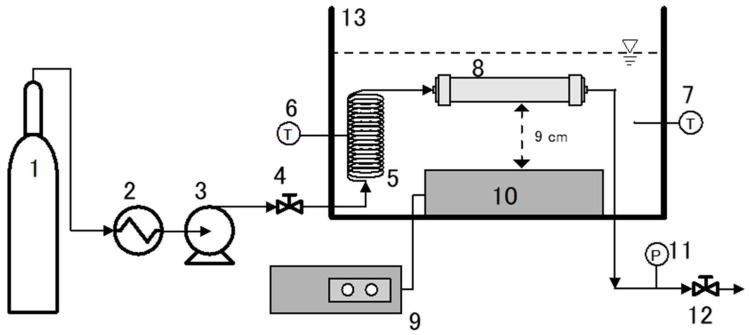
Scheme of the experimental apparatus: 1. CO_2_ cylinder; 2. chiller; 3. high-pressure pump; 4. needle valve; 5. SUS–316 pre-heater; 6, 7. temperature monitor; 8. SUS vessel; 9. ultrasonic controller; 10. ultrasonic vibrator; 11. pressure monitor; 12. BPR; 13. chamber.

Figure 7 shows the scheme of SCCO_2_ in an ultrasound apparatus to generate liposomes from hydrogenated soy phosphatidylcholine. This apparatus comprises a high-pressure CO_2_ pump (PU–2086, Jasco, Tokyo, Japan), ultrasonic equipment (Ultrasonic Multi Cleaner W–118, Honda Electronics Company, Toyohashi, Japan), an acrylic chamber assembled with an electric heater, an SUS vessel (SUS–316; i.d.: 20.0 mm; o.d.: 25.0 mm; length: 250 mm), and a back pressure regulator (BPR; AKICO, Tokyo, Japan) as the main parts. Prior to entering the SUS vessel, CO_2_ was passed through a coil preheater (SUS–316, 1/16 inch, length: 300 cm), which was placed in an acrylic chamber containing liquid water as the heat transfer medium. The preheater was equipped with a K-type thermocouple to determine the experimental temperature. An analog pressure gauge (GLT–21–25 MPa, Migishita Seiki MFG. Co., Ltd., Tokyo, Japan) was also attached between the SUS vessel and the BPR via a 1/16 inch stainless steel tube (SUS–316) to inspect the experimental pressure. The experiments were performed as follows. First, hydrogenated soy phosphatidylcholine (30–60 mg) was loaded and sealed in the SUS vessel with distilled water (60 mL). The sealed SUS vessel was then assembled in the SCCO_2_–ultrasound apparatus, immersed in an acrylic chamber, and heated to 40–70 °C. To achieve the SCCO_2_ conditions, CO_2_ was pumped using a high-pressure pump at 10–25 MPa. Subsequently, it was ultrasonically irradiated at 45 kHz with an input power of 600 W. After 60 min, the pressure of the SCCO_2_–ultrasound apparatus was depressurized and the liposomal dispersion product was collected. Pressure depressurization was performed as follows. The SUS vessel was removed from the acrylic chamber and vertically positioned for approximately 15 min. Subsequently, the pressure was gradually released from the top of the SUS vessel via the BPR device. The same method was applied when the SCCO_2_–ultrasound apparatus was employed to generate curcumin-encapsulating liposomes. Each experiment was conducted in duplicate or triplicate, and the data represent the mean values from the results. The effects of the wall thickness of the SUS vessel on the liposome products were not investigated.

### 3.3. Analytical Methods

The amount of encapsulated curcumin was analyzed using reversed-phase HPLC with a Jasco system comprising a column heater (U–620, Sugai Chemie, Inc., Wakayama, Japan), degasser (DG–980–50, Jasco Co., Inc., Tokyo, Japan), pump (PU-980, Jasco Co., Inc., Tokyo, Japan), and UV–vis detector (UV-970, Jasco Co., Inc., Tokyo, Japan). HPLC was performed and the chromatogram was recorded using a PC driven with Jasco-Borwin (Ver 1.5) software via the LC–Net II/ADC controller (Jasco Co., Tokyo, Japan). Using an Inertsil ODS-3 column (250 mm × 4.6 mm × 5 μm, GL Sciences, Tokyo, Japan), the HPLC analysis system was operated as follows: flow rate: 1.0 mL/min; acetonitrile/water: 90/10 (eluent); UV detector: 420 nm; temperature: 40 °C. The size distribution of the liposome products was measured using DLS (Malvern Instruments, Malvern, Worcestershire, England). The zeta potential was measured in zeta potential measurement mode using a ZetasizerNano ZS instrument (Malvern Instruments Ltd., Worcestershire, UK). The liposomal dispersion product was diluted 10 times with distilled water and introduced into the measuring device with 173° scattering at 25 °C [46]. High-resolution transmission electron microscopy (HRTEM, JEM–2100Plus, JEOL Ltd., Tokyo, Japan) was used to observe the lamellarity and surface of the liposome products. The acceleration voltage was set to 200 kV. Similarly to the size distribution and zeta potential analysis, the liposomal dispersion product was also diluted 10 times with distilled water. It was then treated with a phosphotungstic acid solution (2.0%) to prepare HRTEM negative-stain liposome products. Next, a few drops of the solution were drop-cast onto a copper grid coated with carbon (NP-C15; Okenshoji Co., Ltd., Tokyo, Japan) and stored in a desiccator at room temperature until further analysis (overnight). 

## 4. Conclusions

Curcumin encapsulation using liposomes generated from hydrogenated soy phosphatidylcholine in an ultrasound environment under pressurized carbon dioxide was successfully performed at operating temperatures of 40–70 °C and pressures of 10–25 MPa. Experiments were performed in a batch system with 60 min of ultrasonication. TEM images showed that spherical liposomes with diameters of less than 100 nm were formed. DLS analysis indicated that the curcumin-loaded liposome nanosuspension exhibited good stability. The changing operating conditions seemed to affect the amount of liposome-encapsulated curcumin, where increasing the operating temperature or pressure increased the diameter of the liposome products and decreased the amount of liposome-encapsulated curcumin. Although the application of SCCO_2_ in an ultrasound environment has been demonstrated for curcumin encapsulation using liposomes, certain challenges remain, mainly in relation to the SCCO_2_–ultrasound system being designed for small-scale processes without an agitation tool that may improve the encapsulation efficiency. Nevertheless, this SCCO_2_–ultrasound system is a powerful technique for generating organic-solvent-free phospholipid-based drug-loaded liposomes for industrial applications and may provide information for the generation of liposomes from phospholipids.

## Figures and Tables

**Table 1:** Encapsulated curcumin, liposome particle size, zeta potential, and dispersity index.

Temperature (°C)	Pressure (MPa)	Encapsulated Curcumin (mg)	Diameter (nm)	Zeta Potential (mV)	Dispersity
40	10	0.27	219.27 ± 10.25	−63.40 ± 6.46	0.26 ± 0.04
	25	0.18	814.93 ± 5.42	−55.00 ± 5.86	0.32 ± 0.03
50	10	0.74	290.10 ± 5.52	−63.50 ± 3.82	0.32 ± 0.02
	15	0.53	752.80± 4.67	−76.00 ± 5.03	0.43 ± 0.02
	20	0.40	925.90 ± 6.63	−61.00 ± 3.24	0.44 ± 0.02
	25	0.28	936.60 ± 6.34	−64.80 ± 7.52	0.46 ± 0.01
60	10	0.54	371.27 ± 2.43	−51.80 ± 5.63	0.22 ± 0.01
	25	0.26	1157.67 ± 8.74	−59.90 ± 4.99	0.42 ± 0.02
70	10	0.59	233.10 ± 9.17	−38.10 ± 5.56	0.21 ± 0.03
	15	0.31	646.90 ± 7.26	−45.30 ± 7.96	0.30 ± 0.02
	20	0.19	863.50 ± 8.85	−52.30 ± 5.60	0.37 ± 0.02
	25	0.20	1033.00 ± 6.88	−54.50 ± 4.50	0.41 ± 0.04

## Data Availability

Not applicable.
